# Miscarriage, stillbirth, and mortality risk from stroke in women: findings from the PLCO study

**DOI:** 10.4178/epih.e2024093

**Published:** 2024-11-25

**Authors:** Hui Tang, Zhou Li, Yuan Zhang, Mingjun Dai, Xiaoya Wang, Chuan Shao

**Affiliations:** 1 Department of Neurosurgery, Nanchong Central Hospital, The Second Clinical Medical College, North Sichuan Medical College, Nanchong, China; 2 Nanchong Institute of Cerebrovascular Diseases, Nanchong, China; 3 Sichuan Clinical Research Center for Neurological Disease, Nanchong, China; 4 Department of Pathology, Affiliated Hospital of North Sichuan Medical College, Nanchong, China; 5 Department of Neurosurgery, Chongqing General Hospital, Chongqing University, Chongqing, China

**Keywords:** Miscarriage, Stillbirth, Stroke, Mortality

## Abstract

**OBJECTIVES:**

Existing evidence suggests that miscarriage and stillbirth are associated with an increased risk of stroke in women. However, the impact of these events on stroke mortality remains unclear. This study aimed to elucidate the potential association between miscarriage and stillbirth and stroke mortality in women.

**METHODS:**

We employed a competing risk model using data from the Prostate, Lung, Colorectal, and Ovarian Cancer Screening Trial to assess the relationship between miscarriage/stillbirth and stroke death. Death from other causes was considered as a competing risk, and we conducted a subgroup analysis to explore the potential impact.

**RESULTS:**

Our study included 68,629 women for miscarriage and 65,343 women for stillbirth. No significant association was observed between miscarriage and stroke mortality (hazard ratio [HR], 0.96; 95% confidence interval [CI], 0.84 to 1.10; p=0.58). While a single stillbirth did not show a significant association (HR, 0.81; 95% CI, 0.57 to 1.15; p=0.23), recurrent stillbirth (≥2) was associated with a significantly increased risk of stroke mortality compared to women with no stillbirths (HR, 2.24; 95% CI, 1.45 to 3.46; p<0.001).

**CONCLUSIONS:**

Our findings suggest that recurrent stillbirth, but not single events, is associated with an elevated risk of stroke mortality in women. Further research is warranted to clarify the underlying mechanisms and potential long-term health implications of recurrent pregnancy loss.

## GRAPHICAL ABSTRACT

[Fig f6-epih-46-e2024093]

## Key Message

Recurrent stillbirth is associated with increased stroke mortality in women, suggesting it may be a marker of long-term cerebrovascular risk. Further research is needed to elucidate the underlying mechanisms and develop effective prevention strategies.

## INTRODUCTION

Stroke is a major public health problem. Its incidence rate has risen by 70% over the past 3 decades, while its mortality rate has increased by 43%. These trends contribute to an estimated economic burden surpassing US$891 billion, which accounts for 1.12% of the global gross domestic product [1].

Previous studies have indicated that stroke is the fourth leading cause of death in women (6.2%) and the sixth in men (4.4%) [2]. Women generally have a longer lifespan than men, resulting in a higher proportion of older women. Regrettably, the incidence of stroke rises with age, significantly increasing the risk of stroke-related mortality among elderly women.

Traditional risk factors such as smoking, hypertension, obesity, and diabetes are well-established contributors to stroke in both genderrs. However, recent research has explored women-specific factors, such as complications during pregnancy, that may influence stroke mortality. Miscarriage and stillbirth, which affect approximately 15.3% and 0.35% of pregnancies respectively [3,4], are not only emotionally devastating but may also pose long-term health risks. Emerging evidence indicates a connection between miscarriage and stillbirth and an increased risk of arterial thrombosis, atherosclerosis, and renovascular hypertension—all known contributors to stroke [5–7]. However, the evidence linking these factors to stroke mortality is still inconclusive, with some studies indicating positive associations and others not reaching statistical significance [8–15].

Our study aims to address this critical gap in knowledge by using a competing risk model to explore the potential association between miscarriage or stillbirth and stroke mortality in women, within the context of the Prostate, Lung, Colorectal, and Ovarian (PLCO) Cancer Screening Trial. This robust dataset provides a unique opportunity to assess the long-term effects of pregnancy complications on stroke mortality, while accounting for competing risks and potential confounding factors.

## MATERIALS AND METHODS

### Data set

The PLCO Cancer Screening Trial was a large, randomized, controlled trial designed to assess whether specific cancer screening tests could reduce mortality rates for prostate, lung, colorectal, and ovarian cancers [16,17]. This extensive study enrolled approximately 155,000 participants from November 1993 to July 2001. It was conducted across 10 different centers in the United States, including Birmingham, Detroit, Denver, Honolulu, Marshfield, Minneapolis, Pittsburgh, Salt Lake City, St. Louis, and Washington D.C. Detailed eligibility criteria for the study are available on the National Cancer Institute’s official website (https://cdas.cancer.gov/learn/plco). The trial utilized a randomized design, dividing participants into 2 groups: an intervention arm that received specific cancer screenings and a control arm that followed standard medical practices without additional screenings. Participants provided comprehensive baseline information through questionnaires, which covered various aspects such as demographics, physical characteristics, medical history, lifestyle factors including smoking, and reproductive health details.

For our current research, we utilized data from the PLCO Cancer Screening Trial to explore the relationship between miscarriage, stillbirth, and stroke mortality.

### Participant screening

**Figure 1:**
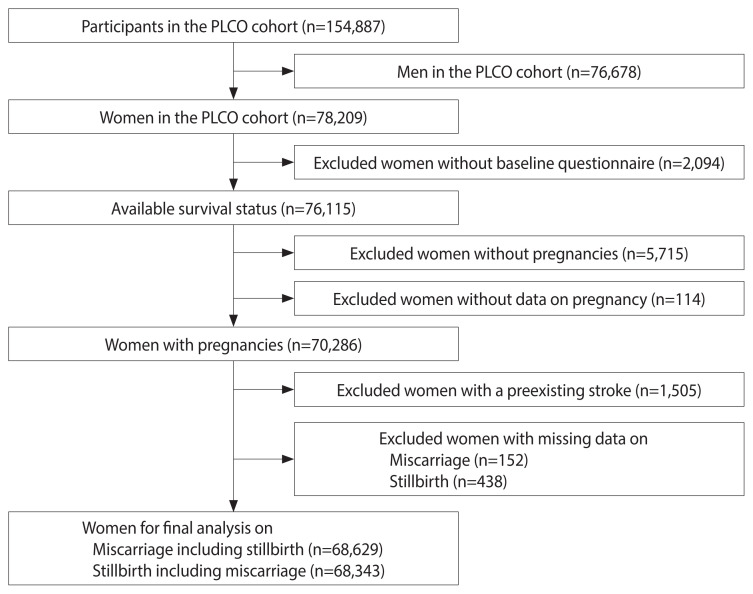
Flow chart of the screening process. PLCO, cohort, Prostate, Lung, Colorectal, and Ovarian Cancer Screening Trial.

According to the study design, 76,678 men participants were initially excluded, while 78,209 women participants were included. Of these, 76,115 women participants completed the baseline questionnaires. A total of 5,715 women were excluded due to not being pregnant, and an additional 114 were excluded due to missing pregnancy data. Furthermore, 1,505 women with a history of stroke, along with 152 lacking information on miscarriage and 438 on stillbirth, were also excluded from all analyses. Ultimately, 68,629 women were included in the study for miscarriage analysis and 68,343 for stillbirth analysis. The screening process is illustrated in Figure 1.

### Exposure assessment

Miscarriages and stillbirths are categorized based on their frequency into 3 groups: 0, 1, and ≥2. Recurrent stillbirths are defined as having occurred 2 or more times, regardless of any intervening live births. Surveys investigating miscarriage and stillbirth in women included questions such as, “How many of your pregnancies resulted in a miscarriage or an abortion?” and “How many of your pregnancies resulted in a stillbirth?”

### Outcome assessment

To ensure complete death certificate collection, the PLCO Screening Centers implemented a multifaceted approach: annual study update forms, obituaries or reports from relatives, and extensive searches of the Social Security Death Index and National Death Index for all deaths up to 2018 [15]. To distinguish deaths caused by stroke from other causes, death certificates from state vital statistics bureaus were examined using the 9th edition of the International Classification of Diseases. This procedure followed the guidelines established by the National Center for Health Statistics. A Death Review Process was also established to confirm the accuracy of the trial outcomes. This process included a thorough review of medical records for all deaths, which was then used in the statistical analysis of the primary endpoints. Survival time was calculated from the date of randomization to either the date of death due to stroke or the cutoff date for follow-up.

### Statistical analysis

All statistical analyses were conducted using R version 4.1.1 (R Foundation for Statistical Computing, Vienna, Austria). Baseline characteristics were presented as numbers and percentages for categorical variables, with differences compared using the chi-square test. Continuous variables were presented as medians (interquartile range), with differences compared using the Kruskal-Wallis rank sum test.

The hazard ratios (HRs) with 95% confidence intervals (CIs) were calculated using the competing risk model (package “cmprsk,” version 2.2–11) to assess the relationship between miscarriage or stillbirth and stroke-related mortality [18].

Women who did not report a miscarriage or stillbirth were designated as the reference group, while those who reported these events were categorized as the exposure group. The primary outcome of interest was defined as death due to stroke, whereas deaths from other causes were regarded as competing events. Initially, we conducted comparisons between “stillbirth versus non-stillbirth” and “miscarriage versus non-miscarriage.” Subsequently, we performed linear trend tests by treating the status of miscarriage and stillbirth (0, 1, and ≥2) as a continuous variable in our models.

Covariates were selected based on prior literature that examined the associations between stroke mortality and a history of adverse pregnancy outcomes, such as miscarriage and stillbirth. The confounding variables adjusted for at baseline included age (continuous), race (white or other), education level (below or at least university), smoking status (never, former, or current smokers), body mass index (BMI: underweight and normal [<25 kg/m^2^], overweight [25–30 kg/m^2^], and obesity [>30 kg/m^2^]), hypertension (yes or no), heart attack (yes or no), diabetes mellitus (yes or no), and study arm (intervention or control).

Since some women may have multiple pregnancy experiences, they may encounter both miscarriage and stillbirth during their reproductive years. In our study, we conducted 2 separate sensitivity analyses. The first, termed “miscarriage alone,” focused exclusively on women without any history of stillbirth. Similarly, the second analysis, “stillbirth alone,” excluded those with any history of miscarriage.

Subgroup analyses were conducted based on various factors including age (<65 and ≥65), race, education level, smoking status, BMI, hypertension, heart attack, and diabetes mellitus to explore the association between miscarriage or stillbirth and stroke mortality. All statistical tests were 2-sided, and a p-value <0.05 was considered statistically significant.

### Ethics statement

The study protocol received approval from the Institutional Review Board of the National Cancer Institute (PLCO-811, PLCO-836). Further approval was not required.

## RESULTS

In the original PLCO Cancer Screening Trial, there were 17,209 recorded deaths among women, which included 1,159 from stroke and 16,050 from other causes (Supplementary Material 1). In this study, 68,629 and 68,343 women were included in the analyses of miscarriage and stillbirth, respectively. The baseline characteristics of the participants are summarized in Table 1 (Supplementary Material 2). The stroke mortality was 1.4% (616/43,221) among women with no miscarriage, 1.4% (223/16,402) among women with 1 miscarriage, and 1.6% (143/9,006) among women with ≥2 miscarriages. However, stroke mortality showed an increase from 1.4% (917/65,123) in women with no stillbirth to 3.7% (23/623) in women with ≥2 stillbirths, with a rate of 1.4% (36/2,597) among women with 1 stillbirth.

### Miscarriage and stroke mortality

No significant difference was found in either the unadjusted model (HR, 1.00; 95% CI, 0.88 to 1.14; p=0.98) or the adjusted model (HR, 0.96; 95% CI, 0.84 to 1.10; p=0.58) when comparing pregnant women with prior miscarriage to those without any history of miscarriages (Table 2, Supplementary Material 3). In a sensitivity analysis, women with a history of stillbirth during the study were excluded. The results were not significantly different (Supplementary Material 4).

**Figure 2:**
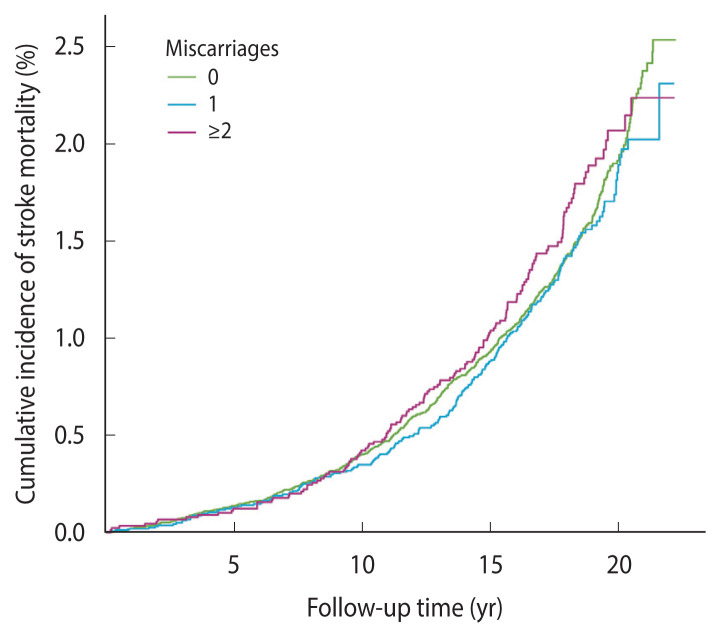
Cumulative incidence curves of stroke mortality for women among with different miscarriage statuses. The curves showed no significant differences in the risk of stroke mortality among women with no miscarriage, 1 miscarriage and ≥2 miscarriages.

**Figure 3:**
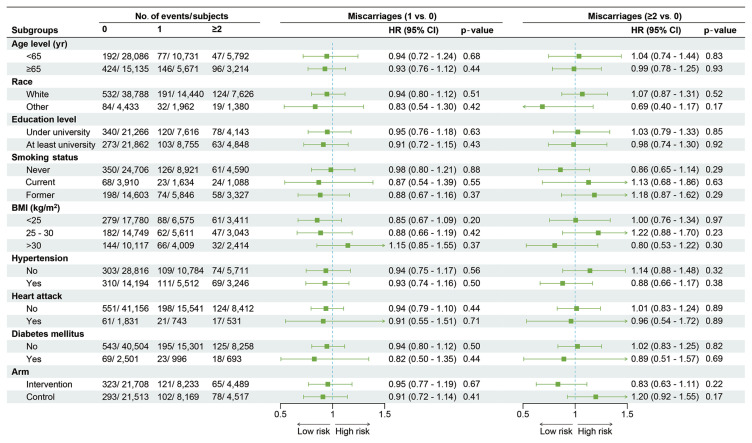
Subgroup analysis and forest plot for stroke mortality in women with different miscarriage statuses. This forest plot shows the number of deaths and subjects, with adjusted HRs and 95% CIs, according to baseline characteristics. HRs were adjusted for age, arm, education level, smoking status, BMI, hypertension, heart attack, diabetes mellitus and race. HR, hazard ratio; CI, confidence interval; BMI, body mass index.

Further analysis showed no significant relationship between the status of miscarriage and stroke mortality risk. The adjusted HRs and 95% CIs were 0.94 (95% CI, 0.80 to 1.10; p=0.42) for women with only 1 miscarriage and 1.01 (95% CI, 0.83 to 1.22; p=0.95) for women with ≥2 miscarriages (Table 2). The cumulative incidence of stroke mortality from baseline to the end of follow-up was similar among women with no miscarriage, 1 miscarriage and ≥2 miscarriages (Figure 2). No significant linear trend was observed between miscarriage status and stroke mortality (p for trend=0.80). Further analysis yielded similar findings across subgroups (Figure 3).

### Stillbirth and stroke mortality

Regarding stillbirths, an analysis of the comparison of “stillbirth versus non-stillbirth” was first performed. Results showed no significantly increased risk of stroke mortality in women with a history of stillbirth (adjusted HR, 1.07; 95% CI 0.81 to 1.41; p=0.63) (Table 2, Supplementary Material 5). A sensitivity analysis was performed with the exclusion of women with miscarriage, and similar findings were also observed (Supplementary Material 4).

**Figure 4:**
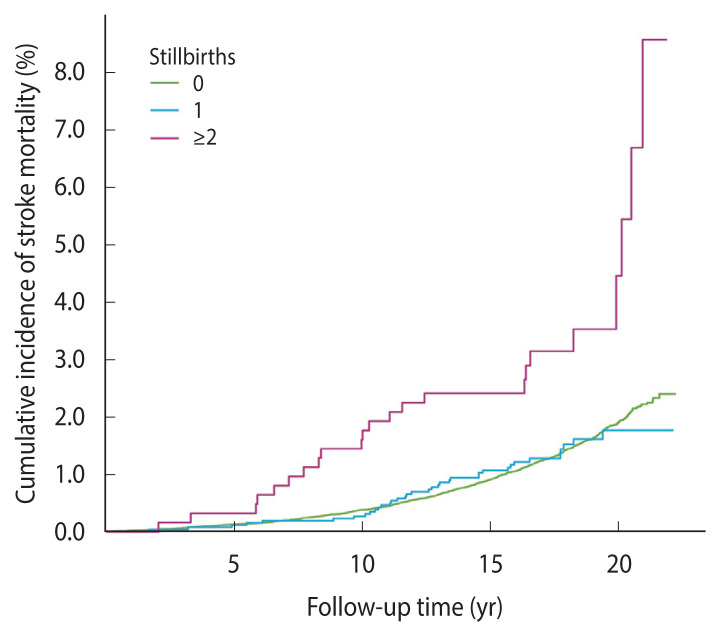
Cumulative incidence curves of stroke mortality among women with different stillbith statuses. The cumulative incidence of stroke mortality was similar between women with no stillbirth and those with 1 stillbirth, but significantly higher among women with ≥2 stillbirths.

**Figure 5:**
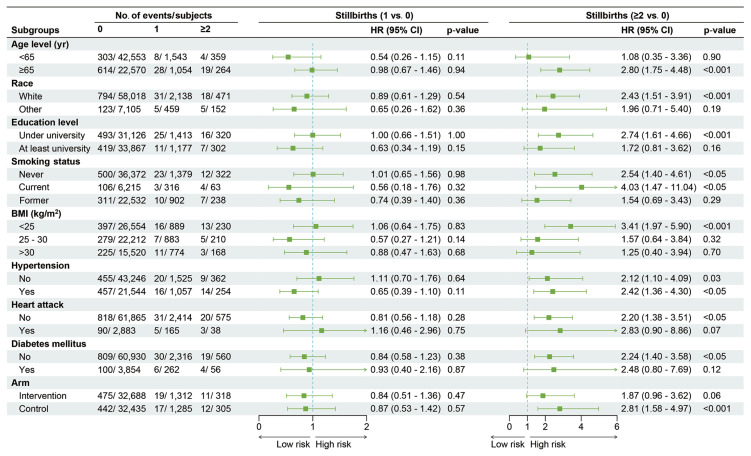
Subgroup analysis and forest plot for stroke mortality in women with different stillbirth statuses. This forest plot shows the number of deaths and subjects, with adjusted HRs and 95% CIs, according to baseline characteristics. HRs were adjusted for age, arm, education level, smoking status, BMI, hypertension, heart attack, diabetes mellitus and race. HR, hazard ratio; CI, confidence interval; BMI, body mass index.

When a dose-exposure analysis of the relationship between the status of stillbirths and stroke risk was performed, a noticeably increased risk was identified in women with ≥2 stillbirths (adjusted HR, 2.24; 95% CI, 1.45 to 3.46; p<0.001), but not in women with only 1 stillbirth (adjusted HR, 0.81; 95% CI, 0.57 to 1.15; p=0.23) (Table 2). Cumulative incidence curves revealed a significantly higher risk of stroke mortality among women with ≥2 stillbirths compared to those with no stillbirths or 1 stillbirth (Figure 4). The linear trend between stillbirth status (0, 1, and ≥2) and stroke mortality did not reach statistical significance (p for trend=0.11). Subgroup analyses revealed low education levels and current smoking are associated with a higher risk of death from stroke (Figure 5).

## DISCUSSION

In the current study, we found an increased risk of stroke mortality was associated with recurrent stillbirths (≥2), but not a single stillbirth or miscarriage.

Evidence for the relationship between stroke mortality and miscarriage was limited. A pooled analysis of 5 studies, which included over 541,379 women, indicated that miscarriage increases the risk of stroke mortality [19]. However, this association was not observed in the current study, which primarily involved white non-Hispanic Americans, making up nearly 90% of the participants. Similarly, no such association was confirmed in a cohort study involving 267,400 Chinese women textile workers [13]. Gallagher et al. [13] reported that neither spontaneous nor induced abortion increased the risk of death from hemorrhagic or ischemic stroke. Several factors could potentially explain this observed disparity. First, the limited statistical power due to the sample size may have influenced the findings in this study and the cohort of Chinese women textile workers. Second, the InterLACE study used Cox proportional hazards models, which might have overestimated the effect of miscarriage on stroke mortality due to the presence of competing risks from other causes of death. Third, the InterLACE study did not consider stillbirth as a potential confounder, which could have affected the observed association between miscarriage and stroke mortality. Consequently, it remains uncertain whether the observed effect of miscarriage on stroke is independent of stillbirth in the InterLACE study.

Epidemiological findings regarding stillbirth and the morbidity and mortality of stroke have not always been consistent [5,8,10,13, 19–23]. The cohort study in China showed that neither a single stillbirth nor multiple stillbirths increased the mortality risk from ischemic or hemorrhagic stroke [13]. Another study reported that women with previous stillbirths faced a slightly higher risk of stroke death compared to those without [19]. Further analysis revealed that women with recurrent stillbirths (≥2) were at an increased risk of dying from stroke, including both ischemic and hemorrhagic types, unlike women with only 1 stillbirth [19]. Our current observations support these findings, indicating a higher risk of stroke death among women with recurrent stillbirths (≥2). The reasons for these disparities remain unclear. Notably, in the cohort of 267,400 Chinese women, 59.3% were under 50 years of age at enrollment, and all were under 65 at the end of the follow-up period. However, approximately three-quarters of all strokes occur in individuals aged 65 years or older [24], which may lead to an underestimation of stroke mortality [25,26]. The results remained stable across various analyses that explored the relationships between miscarriage and stroke mortality with and without stillbirth, as well as between stillbirth and stroke mortality with and without miscarriage. Additionally, lower educational attainment, often linked with lower socioeconomic status, is associated with decreased health literacy and a higher likelihood of engaging in behaviors that increase stroke risk among women [27].

The exact mechanisms linking miscarriage or stillbirth with an increased risk of stroke are not fully understood. However, several hypotheses have been proposed. First, miscarriage or stillbirth may share common predisposing factors with stroke, such as hypertension or cardiovascular diseases, which can contribute to an increased risk of stroke [19]. Second, women who have experienced miscarriages and have antiphospholipid antibody syndrome are prone to thrombosis in small and medium-sized blood vessels due to hypercoagulable factors. This condition can lead to ischemic stroke if the cerebral vessels are involved [7,28,29]. Third, endothelial dysfunction, which contributes to placental defects resulting in miscarriage, may persist after pregnancy. This dysfunction can prevent vasodilation and promote thrombosis, thereby increasing stroke mortality [30]. Finally, women who have experienced stillbirths often suffer from immune dysfunction, such as immune rejection, which may diminish anti-inflammatory effects and increase the mortality from cerebrovascular diseases [31,32].

The strengths of this study included its large prospective cohort and long-term follow-ups. Additionally, multiple outcome events were recorded among the participants, although each participant could only experience death once. Therefore, a competing risk model was utilized to analyze the risk of death from stroke following miscarriage or stillbirth [33]. Nonetheless, our study had some limitations. First, the data on reproductive factors were based on a retrospective and self-reporting questionnaire, which is susceptible to recall bias, particularly regarding sensitive events such as miscarriages and stillbirths. Given the emotional nature of these experiences, participants may underreport such events. Second, we did not perform an analysis to distinguish hemorrhagic from ischemic stroke deaths, since detailed data about stroke subtypes were lacking in the PLCO study. Third, a residual confounding effect could not be excluded, although a number of covariates were controlled in the study. Finally, similar to previous studies [19,23], our study did not assess the relationship between stroke risk and types of abortions (spontaneous vs. induced) and the timing of abortions. Additionally, it is important to note that miscarriage and stillbirth can result from factors involving both genders. Abnormalities in the father’s germ cells may also contribute to abnormal fetal development [4,34].

In conclusion, we found that women who experienced recurrent stillbirths were at an increased risk of dying from a stroke. This suggests that complications during pregnancy could serve as early indicators of a woman’s long-term cerebrovascular health. To substantiate our findings, well-designed prospective cohort studies that utilize validated medical records to assess miscarriages and stillbirths are necessary. Additionally, further research is needed to better understand the underlying mechanisms of these associations and to develop targeted strategies for prevention and early intervention.

## Figures and Tables

**Figure f6-epih-46-e2024093:**
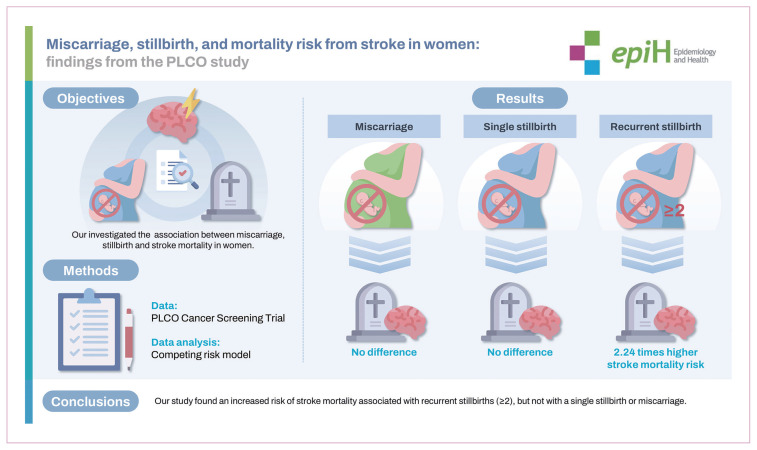


**Table 1:** Baseline characteristics of the subjects by the history of miscarriage and stillbirth at baseline

Characteristics	Miscarriage^1^			p-value^3^	Stillbirth^2^			p-value^3^
	0	1	≥2		0	1	≥2	
Sample size	43,221 (63.0)	16,402 (23.9)	9,006 (13.1)		65,123 (95.3)	2,597 (3.8)	623 (0.9)	
Age (yr)^4^	62 (58, 67)	62 (58, 67)	62 (58, 67)	0.010	62 (58, 67)	63 (59, 68)	63 (59, 68)	<0.001
Race				<0.001				<0.001
White	38,788 (89.7)	14,440 (88.0)	7,626 (84.7)		58,018 (89.1)	2,138 (82.3)	471 (75.6)	
Other	4,433 (10.3)	1,962 (12.0)	1,380 (15.3)		7,105 (10.9)	459 (17.7)	152 (24.4)	
Education level				<0.001				<0.001
Under university	21,266 (49.3)	7,616 (46.5)	4,143 (46.1)		31,126 (47.9)	1,413 (54.6)	320 (51.4)	
At least university	21,862 (50.7)	8,755 (53.5)	4,848 (53.9)		33,867 (52.1)	1,177 (45.4)	302 (48.5)	
Missing	93	31	15		130	7	1	
Smoking status				<0.001				<0.001
Never	24,706 (57.2)	8,921 (54.4)	4,590 (51.0)		36,372 (55.8)	1,379 (53.1)	322 (51.7)	
Former	14,603 (33.8)	5,846 (35.6)	3,327 (36.9)		22,532 (34.6)	902 (34.7)	238 (38.2)	
Current	3,910 (9.0)	1,634 (10.0)	1,088 (12.1)		6,215 (9.5)	316 (12.2)	63 (10.1)	
Missing	2	1	1		4	0	0	
Body mass index (kg/m^2^)				<0.001				<0.001
<25	17,780 (41.7)	6,575 (40.6)	3,411 (38.5)		26,554 (41.3)	889 (34.9)	230 (37.8)	
25–30	14,749 (34.6)	5,611 (34.6)	3,043 (34.3)		22,212 (34.5)	883 (34.7)	210 (34.5)	
>30	10,117 (23.7)	4,009 (24.7)	2,414 (27.2)		15,520 (24.1)	774 (30.4)	168 (27.6)	
Missing	575	207	138		837	51	15	
Hypertension				<0.001				<0.001
No	28,816 (67.0)	10,784 (66.2)	5,711 (63.8)		43,246 (66.7)	1,525 (59.1)	362 (58.8)	
Yes	14,194 (33.0)	5,512 (33.8)	3,246 (36.2)		21,544 (33.2)	1,057 (40.9)	254 (41.2)	
Missing	211	106	49		333	15	7	
Heart attack				<0.001				<0.001
No	41,156 (95.7)	15,541 (95.4)	8,412 (94.1)		61,865 (95.5)	2,414 (93.6)	575 (93.8)	
Yes	1,831 (4.3)	743 (4.6)	531 (5.9)		2,883 (4.4)	165 (6.4)	38 (6.2)	
Missing	234	118	63		375	18	10	
Diabetes mellitus				<0.001				<0.001
No	40,504 (94.2)	15,301 (93.9)	8,258 (92.3)		60,930 (94.0)	2,316 (89.8)	560 (90.9)	
Yes	2,501 (5.8)	996 (6.1)	693 (7.7)		3,854 (5.9)	262 (10.2)	56 (9.1)	
Missing	216	105	55		339	19	7	
Arm				0.800				0.870
Intervention	21,708 (50.2)	8,233 (50.2)	4,489 (49.8)		32,688 (50.2)	1,312 (50.5)	318 (51.0)	
Control	21,513 (49.8)	8,169 (49.8)	4,517 (50.2)		32,435 (49.8)	1,285 (49.5)	305 (49.0)	

Values are presented as number (%).

1 All women who reported a history of miscarriage in the questionnaire were included.

2 All women who reported a history of stillbirth in the questionnaire were included.

3 Pearson’s chi-square test was used to compare categorical variables between groups.

4 For the continuous variable age, which did not meet the assumption of normality as assessed by the Anderson-Darling and Kolmogorov-Smirnov tests, data are presented as median (interquartile range), and the Kruskal-Wallis rank sum test was used to compare median ages across groups.

**Table 2:** Unadjusted and adjusted competing-risk models for stroke mortality

Variables	Events (n)	Subjects (n)	Unadjusted	p-value	Adjusted	p-value
Miscarriage^1^						
Never	616	43,221	1.00 (reference)		1.00 (reference)	
Ever	366	25,408	1.00 (0.88, 1.14)	0.98	0.96 (0.84, 1.10)	0.58
0	616	43,221	1.00 (reference)		1.00 (reference)	
1	223	16,402	0.95 (0.81, 1.10)	0.47	0.94 (0.80, 1.10)	0.42
≥2	143	9,006	1.10 (0.91, 1.31)	0.33	1.01 (0.83, 1.22)	0.95
			p for trend	0.60		0.80
Stillbirth^2^						
Never	917	65,123	1.00 (reference)		1.00 (reference)	
Ever	59	3,220	1.31 (1.01, 1.70)	0.05	1.07 (0.81, 1.41)	0.63
0	917	65,123	1.00 (reference)		1.00 (reference)	
1	36	2,597	0.99 (0.71, 1.38)	0.93	0.81 (0.57, 1.15)	0.23
≥2	23	623	2.67 (1.77, 4.04)	<0.001	2.24 (1.45, 3.46)	<0.001
			p for trend	0.01		0.11

Values are presented as hazard ratio (95% confidence interval).

1 All women who reported a history of miscarriage in the questionnaire were included; Adjusted for age, education level, smoking status, body mass index, history of hypertension, history of heart attack, history of diabetes mellitus, race, arm and stillbirth.

2 All women who reported a history of stillbirth in the questionnaire were included; Adjusted for age, education level, smoking status, body mass index, history of hypertension, history of heart attack, history of diabetes mellitus, race, arm, and miscarriage.

## References

[1] Owolabi MO, Thrift AG, Mahal A, Ishida M, Martins S, Johnson WD (2022). Primary stroke prevention worldwide: translating evidence into action. Lancet Public Health.

[2] Naveed H, Almasri M, Kazani B, Nauman A, Akhtar N, Singh R (2023). Women and stroke: disparities in clinical presentation, severity, and short- and long-term outcomes. Front Neurol.

[3] Arechvo A, Nikolaidi DA, Gil MM, Rolle V, Syngelaki A, Akolekar R (2023). Incidence of stillbirth: effect of deprivation. Ultrasound Obstet Gynecol.

[4] Quenby S, Gallos ID, Dhillon-Smith RK, Podesek M, Stephenson MD, Fisher J (2021). Miscarriage matters: the epidemiological, physical, psychological, and economic costs of early pregnancy loss. Lancet.

[5] Ranthe MF, Andersen EA, Wohlfahrt J, Bundgaard H, Melbye M, Boyd HA (2013). Pregnancy loss and later risk of atherosclerotic disease. Circulation.

[6] Hartasanchez SA, Flores-Torres M, Monge A, Yunes E, Rodriguez B, Cantu-Brito C (2018). Pregnancy loss and carotid intima-media thickness in Mexican women. J Am Heart Assoc.

[7] Maino A, Siegerink B, Algra A, Martinelli I, Peyvandi F, Rosendaal FR (2016). Pregnancy loss and risk of ischaemic stroke and myocardial infarction. Br J Haematol.

[8] Poorthuis MH, Algra AM, Algra A, Kappelle LJ, Klijn CJ (2017). Female- and male-specific risk factors for stroke: a systematic review and meta-analysis. JAMA Neurol.

[9] Stentz NC, Koelper N, Barnhart KT, Sammel MD, Senapati S (2020). Infertility and mortality. Am J Obstet Gynecol.

[10] Liang C, Chung HF, Dobson AJ, Mishra GD (2022). Infertility, miscarriage, stillbirth, and the risk of stroke among women: a systematic review and meta-analysis. Stroke.

[11] Udell JA, Lu H, Redelmeier DA (2013). Long-term cardiovascular risk in women prescribed fertility therapy. J Am Coll Cardiol.

[12] Yamada K, Iso H, Cui R, Tamakoshi A (2017). Recurrent pregnancy loss and cardiovascular disease mortality in Japanese women: a population-based, prospective cohort study. J Stroke Cerebrovasc Dis.

[13] Gallagher LG, Davis LB, Ray RM, Psaty BM, Gao DL, Checkoway H (2011). Reproductive history and mortality from cardiovascular disease among women textile workers in Shanghai, China. Int J Epidemiol.

[14] Skåra KH, Åsvold BO, Hernáez Á, Fraser A, Rich-Edwards JW, Farland LV (2022). Risk of cardiovascular disease in women and men with subfertility: the Trøndelag Health Study. Fertil Steril.

[15] Bungum AB, Glazer CH, Arendt LH, Schmidt L, Pinborg A, Bonde JP (2019). Risk of hospitalization for early onset of cardiovascular disease among infertile women: a register-based cohort study. Hum Reprod.

[16] National Cancer Institute PLCO.

[17] Zhu CS, Pinsky PF, Kramer BS, Prorok PC, Purdue MP, Berg CD (2013). The prostate, lung, colorectal, and ovarian cancer screening trial and its associated research resource. J Natl Cancer Inst.

[18] Fine JP, Gray RJ (1999). A proportional hazards model for the subdistribution of a competing risk. J Am Stat Assoc.

[19] Liang C, Chung HF, Dobson AJ, Hayashi K, van der Schouw YT, Kuh D (2022). Infertility, recurrent pregnancy loss, and risk of stroke: pooled analysis of individual patient data of 618 851 women. BMJ.

[20] Kyriacou H, Al-Mohammad A, Muehlschlegel C, Foster-Davies L, Bruco ME, Legard C (2022). The risk of cardiovascular diseases after miscarriage, stillbirth, and induced abortion: a systematic review and meta-analysis. Eur Heart J Open.

[21] Peters SA, Yang L, Guo Y, Chen Y, Bian Z, Tian X (2017). Pregnancy, pregnancy loss, and the risk of cardiovascular disease in Chinese women: findings from the China Kadoorie Biobank. BMC Med.

[22] Parker DR, Lu B, Sands-Lincoln M, Kroenke CH, Lee CC, O’Sullivan M (2014). Risk of cardiovascular disease among postmenopausal women with prior pregnancy loss: the women’s health initiative. Ann Fam Med.

[23] Peters SA, Woodward M (2018). Women’s reproductive factors and incident cardiovascular disease in the UK Biobank. Heart.

[24] Yousufuddin M, Young N (2019). Aging and ischemic stroke. Aging (Albany NY).

[25] Calderon-Margalit R, Friedlander Y, Yanetz R, Deutsch L, Manor O, Harlap S (2007). Late stillbirths and long-term mortality of mothers. Obstet Gynecol.

[26] Jiang L, Huang S, Hee JY, Xin Y, Zou S, Tang K (2023). Pregnancy loss and risk of all-cause mortality in Chinese women: findings from the China Kadoorie biobank. Int J Public Health.

[27] Che B, Shen S, Zhu Z, Wang A, Xu T, Peng Y (2020). Education level and long-term mortality, recurrent stroke, and cardiovascular events in patients with ischemic stroke. J Am Heart Assoc.

[28] Svenungsson E, Antovic A (2020). The antiphospholipid syndrome - often overlooked cause of vascular occlusions?. J Intern Med.

[29] Feldmann E, Levine SR (1995). Cerebrovascular disease with antiphospholipid antibodies: immune mechanisms, significance, and therapeutic options. Ann Neurol.

[30] Davis CM, Fairbanks SL, Alkayed NJ (2013). Mechanism of the sex difference in endothelial dysfunction after stroke. Transl Stroke Res.

[31] Melzer K, Schutz Y, Boulvain M, Kayser B (2010). Physical activity and pregnancy: cardiovascular adaptations, recommendations and pregnancy outcomes. Sports Med.

[32] Sherer ML, Posillico CK, Schwarz JM (2018). The psychoneuroimmunology of pregnancy. Front Neuroendocrinol.

[33] Austin PC, Lee DS, Fine JP (2016). Introduction to the analysis of survival data in the presence of competing risks. Circulation.

[34] du Fossé NA, van der Hoorn MP, van Lith JM, le Cessie S, Lashley EE (2020). Advanced paternal age is associated with an increased risk of spontaneous miscarriage: a systematic review and meta-analysis. Hum Reprod Update.

