# Supplementary material for: Miscarriage, stillbirth, and mortality risk from stroke in women: findings from the PLCO study
Source: Epidemiol Health. 2024 Nov 25;46:e2024093. doi: 10.4178/epih.e2024093 (PMC11840407; doi:10.4178/epih.e2024093)
Supplement: Supplementary file 1 [file epih-46-e2024093-Supplementary-1.docx]

**Supplementary materials**

**Supplementary Material 1. A summary table of all-causes and specific causes of death in the original PLCO Cancer Screening Trial**

| **Causes of death in women** | **Number of deaths (%)** |
| --- | --- |
| Total deaths | 17,209 (100) |
| Stroke | 1,159 (6.74) |
| Accident | 573 (3.33) |
| Cancer | 5,540 (32.19) |
| Digestive disease | 507 (2.95) |
| Other diseases of the nervous system | 1,028 (5.97) |
| Endocrine, nutritional, metabolic diseases and immunity disorders | 583 (3.39) |
| Infectious disease | 363 (2.11) |
| Ischemic heart disease | 1,673 (9.72) |
| Other circulatory diseases | 2,272 (13.20) |
| Respiratory illness | 1,656 (9.62) |
| Other | 1,855 (10.78) |

The causes of death among women were categorized into eleven distinct groups based on the International Classification of Diseases, Ninth Edition. Data are presented as numbers (%). PLCO, Prostate, Lung, Colorectal, and Ovarian.
