# Supplementary material for: Miscarriage, stillbirth, and mortality risk from stroke in women: findings from the PLCO study
Source: Epidemiol Health. 2024 Nov 25;46:e2024093. doi: 10.4178/epih.e2024093 (PMC11840407; doi:10.4178/epih.e2024093)
Supplement: Supplementary file 2 [file epih-46-e2024093-Supplementary-2.docx]

**Supplementary Material 2. Baseline characteristics of the subjects by the history of miscarriage and stillbirth alone at baseline**

| **Characteristic** | **Miscarriage alone ^*^** | | | ***p* value** | **Stillbirth alone ^#^** | | | ***p* value** |
| --- | --- | --- | --- | --- | --- | --- | --- | --- |
|  | **0** | **1** | **≥2** |  | **0** | **1** | **≥2** |  |
| **Sample size** | 41,737 | 15,269 | 8,079 |  | 41,737 | 1,196 | 260 |  |
| **Age (year) ^§^** | 62 (58, 67) | 62 (58, 66) | 62 (58, 66) | 0.02 | 62 (58, 67) | 63 (59, 68) | 62 (58, 67) | <0.001 |
| **Race** |  |  |  | <0.001 |  |  |  | <0.001 |
| **White** | 37,557 (89.98) | 13,529 (88.60) | 6,905 (85.47) |  | 37,557 (89.98) | 1,002 (83.78) | 210 (80.77) |  |
| **Other** | 4,180 (10.02) | 1,740 (11.40) | 1,174 (14.53) |  | 4,180 (10.02) | 194 (16.22) | 50 (19.23) |  |
| **Education level** |  |  |  | <0.001 |  |  |  | <0.001 |
| **Under university** | 20,458 (49.12) | 7,001 (45.92) | 3,652 (45.29) |  | 20,458 (49.12) | 676 (56.71) | 116 (44.79) |  |
| **At least university** | 21,191 (50.88) | 8,244 (54.08) | 4,412 (54.71) |  | 21,191 (50.88) | 516 (43.29) | 143 (55.21) |  |
| **Missing** | 88 | 24 | 15 |  | 88 | 4 | 1 |  |
| **Smoking Status** |  |  |  | <0.001 |  |  |  | 0.01 |
| **Never** | 23,889 (57.24) | 8,327 (54.54) | 4,132 (51.15) |  | 23,889 (57.24) | 666 (55.69) | 137 (52.69) |  |
| **Former** | 14,105 (33.80) | 5,433 (35.58) | 2,981 (36.90) |  | 14,105 (33.80) | 389 (32.53) | 97 (37.31) |  |
| **Current** | 3,741 (8.96) | 1,508 (9.88) | 965 (11.95) |  | 3,741 (8.96) | 141 (11.79) | 26 (10.00) |  |
| **Missing** | 2 | 1 | 1 |  | 2 | 0 | 0 |  |
| **BMI (kg/m^2^)** |  |  |  | <0.001 |  |  |  | <0.001 |
| **<25** | 17,237 (41.85) | 6,197 (41.06) | 3,108 (39.01) |  | 17,237 (41.85) | 421 (35.89) | 115 (44.92) |  |
| **25~30** | 14,260 (34.62) | 5,207 (34.50) | 2,737 (34.35) |  | 14,260 (34.62) | 400 (34.10) | 81 (31.64) |  |
| **>30** | 9,695 (23.54) | 3,687 (24.43) | 2,122 (26.63) |  | 9,695 (23.54) | 352 (30.01) | 60 (23.44) |  |
| **Missing** | 545 | 178 | 112 |  | 545 | 23 | 4 |  |
| **Hypertension** |  |  |  | <0.001 |  |  |  | <0.001 |
| **No** | 27,911 (67.20) | 10,125 (66.70) | 5,191 (64.56) |  | 27,911 (67.20) | 720 (60.45) | 168 (65.12) |  |
| **Yes** | 13,622 (32.80) | 5,054 (33.30) | 2,849 (35.44) |  | 13,622 (32.80) | 471 (39.55) | 90 (34.88) |  |
| **Missing** | 204 | 90 | 39 |  | 204 | 5 | 2 |  |
| **Heart attack** |  |  |  | <0.001 |  |  |  | <0.001 |
| **No** | 39,770 (95.80) | 14,489 (95.53) | 7,573 (94.29) |  | 39,770 (95.80) | 1,114 (93.53) | 246 (96.09) |  |
| **Yes** | 1,743 (4.20) | 678 (4.47) | 459 (5.71) |  | 1,743 (4.20) | 77 (6.47) | 10 (3.91) |  |
| **Missing** | 224 | 102 | 47 |  | 224 | 5 | 4 |  |
| **Diabetes mellitus** |  |  |  | <0.001 |  |  |  | <0.001 |
| **No** | 39,165 (94.31) | 14,276 (94.04) | 7,457 (92.77) |  | 39,165 (94.31) | 1,072 (90.08) | 243 (94.19) |  |
| **Yes** | 2,364 (5.69) | 904 (5.96) | 581 (7.23) |  | 2,364 (5.69) | 118 (9.92) | 15 (5.81) |  |
| **Missing** | 208 | 89 | 41 |  | 208 | 6 | 2 |  |
| **Arm** |  |  |  | 0.60 |  |  |  | 0.45 |
| **Intervention** | 20,992 (50.30) | 7,662 (50.18) | 4,014 (49.68) |  | 20,992 (50.30) | 583 (48.75) | 125 (48.08) |  |
| **Control** | 20,745 (49.70) | 7,607 (49.82) | 4,065 (50.32) |  | 20,745 (49.70) | 613 (51.25) | 1. 1.92) |  |

*Women who reported a history of miscarriage in the questionnaire were included, except for those who also reported a history of stillbirth. # Women who reported a history of stillbirth in the questionnaire were included, except for those who also reported a history of miscarriage. §For the continuous variable age, which did not meet the assumption of normality as assessed by the Anderson-Darling and Kolmogorov-Smirnov tests, data are presented as median (interquartile range), the Kruskal-Wallis rank sum test was used to compare median ages across groups. For categorical variables, data are presented as numbers or percentages, Pearson's chi-squared test was used to compare categorical variables between groups. BMI, body mass index.
