# Supplementary material for: Miscarriage, stillbirth, and mortality risk from stroke in women: findings from the PLCO study
Source: Epidemiol Health. 2024 Nov 25;46:e2024093. doi: 10.4178/epih.e2024093 (PMC11840407; doi:10.4178/epih.e2024093)
Supplement: Supplementary file 4 [file epih-46-e2024093-Supplementary-4.docx]

**Supplementary Material 4. Sensitivity analyses**

| **Variables** | **Events (*n*)** | **Subjects (*n*)** | **Unadjusted HR (95% CI)** | ***p* value** | **Adjusted HR (95% CI)** | ***p* value** |
| --- | --- | --- | --- | --- | --- | --- |
| **Miscarriage alone ^*^** |  |  |  |  |  |  |
| **Never** | 591 | 41,737 | Reference |  | Reference |  |
| **Ever** | 326 | 23,348 | 0.98 (0.85–1.12) | 0.72 | 0.96 (0.84–1.11) | 0.61 |
| **0** | 591 | 41,737 | Reference |  | Reference |  |
| **1** | 202 | 15,269 | 0.93 (0.79–1.09) | 0.35 | 0.93 (0.79–1.10) | 0.40 |
| **≥2** | 124 | 8,079 | 1.07 (0.88–1.30) | 0.51 | 1.02 (0.84–1.25) | 0.82 |
|  |  |  | *p* value for trend | 0.87 |  | 0.88 |
| **Stillbirth alone ^#^** |  |  |  |  |  |  |
| **Never** | 591 | 41,737 | Reference |  | Reference |  |
| **Ever** | 25 | 1,456 | 1.23 (0.82–1.84) | 0.31 | 1.08 (0.73–1.62) | 0.70 |
| **0** | 591 | 41,737 | Reference |  | Reference |  |
| **1** | 17 | 1,196 | 1.02 (0.63–1.66) | 0.93 | 0.88 (0.54–1.43) | 0.60 |
| **≥2** | 8 | 260 | 2.17 (1.08–4.36) | 0.03 | 2.12 (1.06–4.24) | 0.03 |
|  |  |  | *p* value for trend | 0.13 |  | 0.32 |

*Women who reported a history of miscarriage in the questionnaire were included, except for those who also reported a history of stillbirth. Adjusted for age, education level, smoking status, body mass index, history of hypertension, history of heart attack, history of diabetes mellitus, race, arm. #Women who reported a history of stillbirth in the questionnaire were included, except for those who also reported a history of miscarriage. Adjusted for age, education level, smoking status, body mass index, history of hypertension, history of heart attack, history of diabetes mellitus, race, arm and miscarriage. *p* < 0.05 was considered statistically significant. HR, hazard ratio; CI, confidence interval.
