# Supplementary material for: Miscarriage, stillbirth, and mortality risk from stroke in women: findings from the PLCO study
Source: Epidemiol Health. 2024 Nov 25;46:e2024093. doi: 10.4178/epih.e2024093 (PMC11840407; doi:10.4178/epih.e2024093)
Supplement: Supplementary file 5 [file epih-46-e2024093-Supplementary-5.docx]

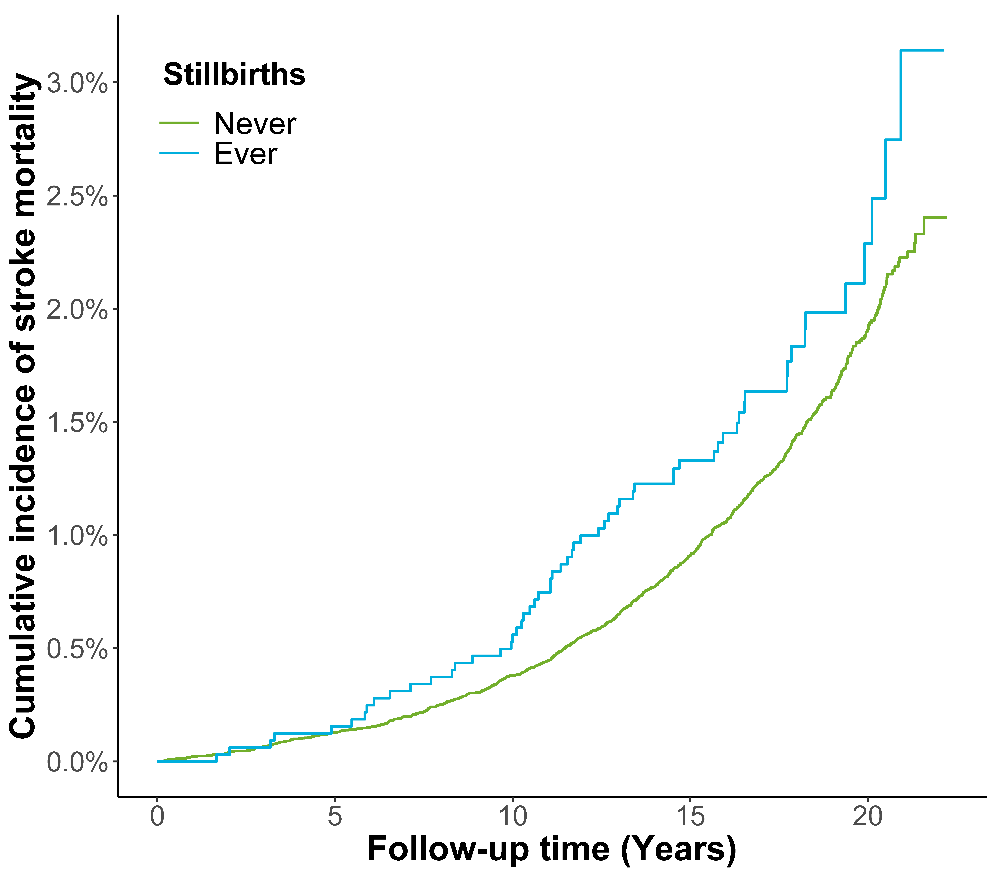


**Supplementary Material 5.** Cumulative incidence curves of stroke mortality among women with and without a history of stillbirth. The resulting curves showed no significant difference in the risk of stroke mortality between women with and without stillbirth.
